# Movement Optimization for a Cyborg Cockroach in a Bounded Space Incorporating Machine Learning

**DOI:** 10.34133/cbsystems.0012

**Published:** 2023-03-15

**Authors:** Mochammad Ariyanto, Chowdhury Mohammad Masum Refat, Kazuyoshi Hirao, Keisuke Morishima

**Affiliations:** ^1^ Department of Mechanical Engineering, Graduate School of Engineering, Osaka University, Suita 565-0871, Japan.; ^2^ Department of Mechanical Engineering, Faculty of Engineering, Diponegoro University, Semarang, 50275, Indonesia.

## Abstract

Cockroaches can traverse unknown obstacle-terrain, self-right on the ground and climb above the obstacle. However, they have limited motion, such as less activity in light/bright areas and lower temperatures. Therefore, the movement of the cyborg cockroaches needs to be optimized for the utilization of the cockroach as a cyborg insect. This study aims to increase the search rate and distance traveled by cockroaches and reduce the stop time by utilizing automatic stimulation from machine learning. Multiple machine learning classifiers were applied to classify the offline binary classification of the cockroach movement based on the inertial measuring unit input signals. Ten time-domain features were chosen and applied as the classifier inputs. The highest performance of the classifiers was implemented for the online motion recognition and automatic stimulation provided to the cerci to trigger the free walking motion of the cockroach. A user interface was developed to run multiple computational processes simultaneously in real time such as computer vision, data acquisition, feature extraction, automatic stimulation, and machine learning using a multithreading algorithm. On the basis of the experiment results, we successfully demonstrated that the movement performance of cockroaches was importantly improved by applying machine learning classification and automatic stimulation. This system increased the search rate and traveled distance by 68% and 70%, respectively, while the stop time was reduced by 78%.

## Introduction

Researchers have utilized insects as hybrid robot platforms [1–7]. These hybrid robots provide advantages such as no need to develop the mechanical robot on a centimeter scale. They possess great locomotion ability, making it difficult for a mechanical robot to compete. Researchers can benefit from their agile locomotion as a cyborg platform for search and rescue missions. Moreover, they can traverse unknown obstacle terrain, avoid the obstacle autonomously without closed-loop feedback control, and follow the wall/edge of the large-size obstacle. One of their great locomotion abilities is that they can self-right on the ground when they are overturned using different strategies [8,9]. They can climb above the obstacle at the height of around 10 mm above the top surface of an obstacle, as performed by Tran-Ngoc et al. [1]. These great locomotion abilities make these hybrid robots suitable for search and rescue missions where they will walk in unknown and unstructured environments.

However, cockroaches are nocturnal animals that are more active at night or in the dark. Previous research shows that they are attracted to dark shelters [10]. After reaching the darkened space, they tend not to move for some period of time. On some occasions, they are lazy to move from one place to another or tend to stay still, especially in the corner area. They have insect latency and corner running behavior [11]. Hence, they like to move in narrow spaces over large spaces because of the latency behavior [12] and stop moving when they get into the narrow space. Another behavior is corner phototaxis. It is a habit of preferring to run along walls and obstacles [13]. The movement of the cockroaches, especially Madagascar hissing cockroach (MHC), is active at higher temperature (80 °F) but sluggish and inactive at the lower temperature (70 °F or lower) [14]. They stop moving when they find a comfortable place to stay. These natural behaviors will hinder the cockroaches to be utilized in unknown and under-rubble environments for search and rescue applications. It will be difficult to apply a mini live stream camera attached to them in a dark or without light areas for real-time monitoring purpose.

Therefore, their movement needs to be optimized for using cyborg insects in various situations. Researchers apply a camera to track the trajectory and the motion of the cyborg insects [1,4–6,15–17]. Their location and trajectory can be obtained using image processing technique. However, using a camera will make their motion (linear acceleration and angular rate) difficult to measure accurately. Moreover, using a camera to obtain insect motion will be challenging, especially in dark under-rubble or sheltered areas. Researchers have used an inertial measuring unit (IMU) to capture the linear acceleration and angular velocity of cyborg insects, as performed by previous studies [1,3,18,19]. Because of its small size, it can be attached to cyborg insects, and the IMU data can be sent to the computer wirelessly. Cole et al. [19] proposed an insect localization technique based on a data-driven inertial navigation system. IMU signals allow the location of the cockroach to be computed where Global Positioning System is not available. This method is restricted to 2-dimensional environments. Tran-Ngoc et al. [1] developed a predictive feedback algorithm to provide steering and acceleration stimulus to the cyborg insect based on the motion signals from IMU. The predictive feedback observed the cockroach linear acceleration and angular velocity and provided stimulus if an obstacle or environment halted the cyborg cockroach. Cole et al. [20] studied offline motion mode recognition to detect the movement of the cyborg equipped with IMU on the electronic backpack. Their study showed that the motion of the cockroach could be distinguished with high accuracy in the offline classification system. Currently, the online classification method implementing machine learning and stimulation feedback based on motion identification has not yet been studied extensively.

This study aims to optimize cockroach movement performance. The cockroach motion measured by IMU will be utilized as the raw input signal for machine learning. It classifies its motion and provides automatic stimulation to the cyborg cockroach in real time. Measured cockroach motions from IMU in time-series data are transferred wirelessly to the desktop PC for real-time computation. This method is implemented to increase the search rate and distance traveled by cockroaches and decrease the cockroach’s stop time in bounded space. This automatic stimulation feedback from machine learning will minimize the given stimulation, optimize the movement, and prevent the cockroaches from fatigue due to too many stimulations.

This study proposes an online classifier and automatic feedback stimulation method based on the IMU signals. IMU has been widely used and integrated with an electronic backpack to measure cyborg insect motion [1,3,18–21]. However, IMU generates bias and noise that will be challenging to process for motion identification of the cockroach. Bias and noise affect and degrade the IMU performance [22,23]. Hence, a machine learning technique is augmented to detect the real-time cockroach movement state from the obtained IMU signals. A custom user interface (UI) is developed using a multithreading algorithm to run multiple computational processes simultaneously in real time such as computer vision, data acquisition, stimulation command, feature extraction, and machine learning classification on a desktop PC. Machine learning is implemented in the online classification of the cockroach cyborg system. The stimulation feedback from the classifier will be given to the cockroach to optimize the movement performance with or without stimulated response. The movement performance metrics in the experimental field for the cockroach will be formulated in Methods.

## Materials and Methods

### Cyborg insect

**Fig. 1.**
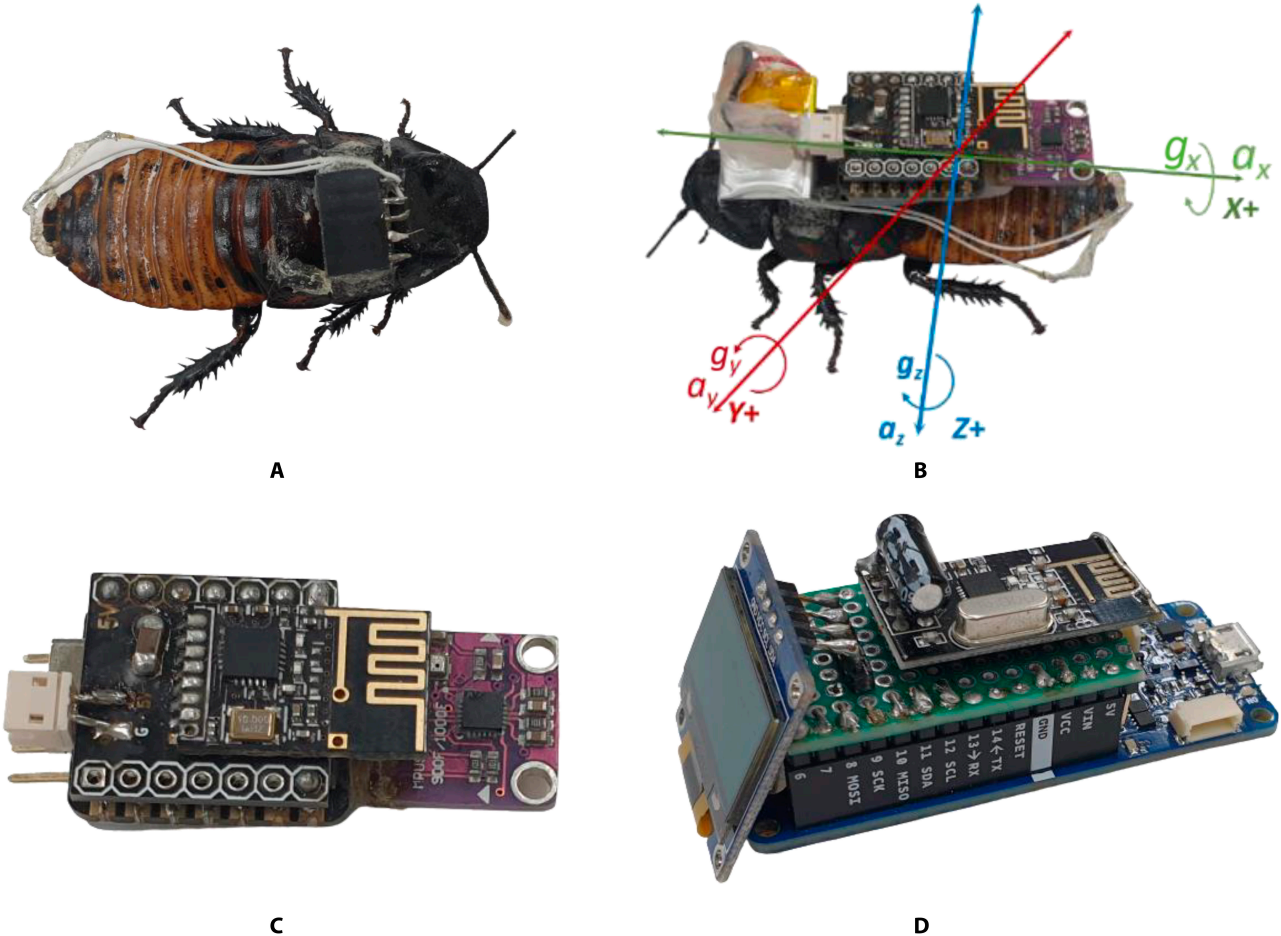
Proposed cyborg and hardware. (A) Implanted electrode on cerci and thorax. (B) Body frame coordinate of the cockroach. (C) Electronic backpack. (D) Wireless transceiver.

In this study, MHC was selected as the cyborg platform because of its bigger size compared to other cockroaches. The size can reach from 5 to 7 cm as an adult cockroach. The cockroaches were treated in the laboratory once a week by cleaning the container and providing new food and water. A platinum wire with a diameter of 0.127 mm was selected as the electrode. Before surgical electrode implantation into cerci, the cockroaches were submerged in small chunks of ice for 30 min for the anesthetization. After 30 min had passed, the cockroach slept for around 10 to 15 min. This sleep time was sufficient for surgical implantation on the cerci and thorax of the cockroach. The platinum electrodes were implanted in the thorax, right cerci, and left cerci. The tips of the cerci were cut around 1 mm from the tip to make a small opening for the electrode. The electrode was inserted into the cerci around 3 mm from the tip of the cerci. The ground electrode was inserted into the middle of the cockroach thorax at a depth of 3 mm. We selected a pin header 2.54-mm female 5-pin single-row strip as the connector between the cockroach and electronic backpack. The connector was glued to the cockroach on the first segment of the thorax. Tinned soft copper wire (single wire) with a diameter of 0.26 mm was utilized as wire extension from the implanted platinum to the header pin connector. The cockroach with the implanted electrode and attached connector is shown in Fig. 1A. Three male cockroaches were implanted with platinum electrodes and utilized in this study. The cockroaches were put back in the container for resting and recovering for more than 24 hours before they could be utilized in the experiments.

The proposed electronic backpack consists of 9-degree-of-freedom IMU sensor, wireless transceiver, and 32-bit microcontroller. A 50-mAh lithium polymer (LiPO) battery was selected to supply power to the electronic backpack due to its lightweight (1.4 g). It can provide power for the electronic backpack for more than 50 min. The total weight of the electronic backpack, excluding the battery, is 5.97 g. The cockroach attached to an electronic backpack and the IMU body coordinate system is shown in Fig. 1B. In this study, the accelerations of the cockroach (longitudinal acceleration *a_x_*, lateral acceleration *a_y_*, and vertical acceleration *a_z_*) are measured by an accelerometer, while the angular rates (roll rate *g_x_*, pitch rate *g_y_*, and yaw rate *g_z_*) are measured using a gyroscope with the coordinate system as shown in Fig. 1B. These signals measured by IMU are transmitted wirelessly to the desktop PC for feature extraction and online machine learning classifier.

### Hardware

A powerful 32-bit 48-MHz microcontroller (SAMD21G18) from Seeed Studio was chosen as the main computation for the electronic backpack. The board size is 20 mm in length and 17.5 mm in width, making it suitable for the cyborg cockroach backpack. The microcontroller is also compatible with Arduino Integrated Development Environment programming. For the wireless transceiver, NRF24L01 surface mount device 2.4-GHz wireless module from Nordic Semiconductor was opted because of its low power consumption. It can communicate wirelessly to transmit the IMU data and receive stimulation commands from the PC. MPU-9250 and BMP280 were implemented as sensors for measuring the cockroach’s kinematics, barometric pressure, and ambient temperature. MPU-9250 consists of a 3-axis gyroscope, 3-axis accelerometer, and 3-axis magnetic field. In this study, only measured accelerations and angular rates from the accelerometer and gyroscope were used for the online motion classification of the cockroach. A custom surface mount device board was developed to integrate the microcontroller and the wireless module, as shown in Fig. 1C. A wireless transceiver was developed to obtain IMU data from the backpack and sent a stimulation command from a desktop PC to the backpack. Arduino MKR Zero was applied as the central controller for the wireless transceiver with NRF24L01 2.4-GHz wireless modules, as shown in Fig. 1D. For the stimulation signal, 50-Hz pulse width modulation signal with a 50% duty cycle was generated using a custom interrupt service routine function under Arduino programming.

### Software

**Fig. 2.**
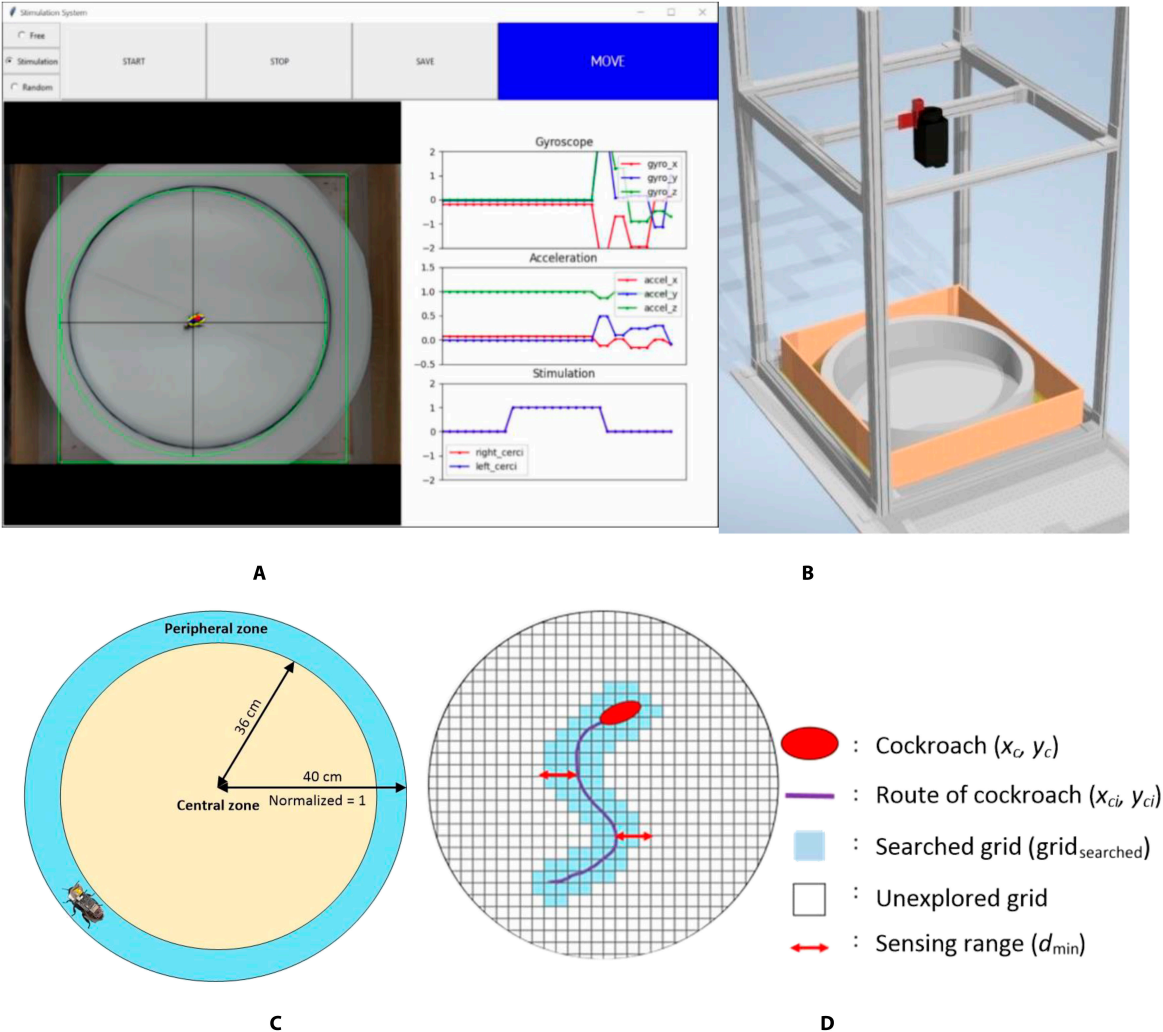
Cockroach in the bounded space arena. (A) Developed UI. (B) Experimental testbed. (C) Central zone and peripheral zone. (D) Proposed model exploration of the MHC.

A custom UI was developed for the interactive and intuitive interface between the desktop PC and the cyborg cockroach in the circular bounded space. Tkinter library was utilized to build the UI. It also managed the overall computational system, such as computer vision, data acquisition, stimulation command, feature extraction, and machine learning classification. A multithreading algorithm was implemented under the threading library in Python programming to run multiple computations simultaneously in real time. A high-performance desktop PC (11th Gen Intel Core i7-11700 @ 2.50-GHz processor and 16-GB of random-access memory) was utilized to run the UI. A web camera from Logitech was placed above the circular arena at a distance of 122 cm. The obtained video from a web camera was processed in real time at 25 frames/s to calculate the position of the cockroaches. The image was filtered using a Gaussian filter, and a Canny edge detector was used to obtain the cockroach edge image. It was processed by mathematical morphology to refine the edge image. Finally, ellipse fitting was chosen and implemented to calculate the position of the cockroach center of mass. The processed cockroach position and trajectory were displayed on the UI in real time. The motion classification result identified from selected online machine learning was displayed on the UI in real time, as shown in Fig. 2A.

In this study, a circular arena with a diameter of 80 cm, as depicted in Fig. 2B, was designed as the experimental arena for the cyborg cockroach. A web camera was installed directly above the circular arena. The camera was connected to a desktop PC running a UI, to capture the position and trajectory of the cyborg cockroach. Jeanson et al. [24] studied German cockroach (*Blattella germanica*) movements in circular bounded space. They developed a numerical movement distribution model of the cockroach derived from their experiments using German cockroaches. The cockroach’s movement most likely accounts for wall following combined with a diffusive random walk.

This research uses peripheral and central zone terms adopted from the previous study [24] in the circular arena. The peripheral zone is a space around walls and obstacles of the circular arena, while the central zone is an open space without walls and obstacles. The peripheral and central zone areas in the circular field are shown in Fig. 2C. We selected the width of the peripheral zone as 4 cm for the MHC. We observed that the MHC could walk freely for the wall following movement in the peripheral area. A previous study showed that the movement of the German cockroach could be classified into several types of movement patterns according to the zone in which the cockroach moved [24]. The movements of the cockroach are listed in Table 1. A random walk is a movement that repeats the movement in irregular directions. Wall following is movement along a wall or obstacle, while the exit is movement from the peripheral zone to the central zone away from the wall or obstacle. In addition, the cockroaches do stop action with a certain probability. The cockroaches prefer to sense the wall with their antennae and run along the wall due to their corner running ability. However, they move irregularly in a space without a wall. On the basis of the previous study for the German cockroach, we observed that MHC performed similar movements to the German cockroaches in the circular bounded space. MHC and German cockroaches performed random walk in the central zone and wall following in the peripheral zone.

**Table 1.** Movement of German cockroaches in the circular bounded space [24].

Zone	Motion mode	Movement
Central zone	Random walk	Move
	Stop	Stop
Peripheral zone	Wall following	Move
	Stop	Stop
	Exit	Move

It is necessary to determine an index score showing the degree of search in the circular bounded space to evaluate the cockroach movement performance. The circular area was divided into a closed area of grids with the same distance for all girds, as shown in Fig. 2D. The range of the circular arena was normalized between −1 and 1. Furthermore, a virtual sensor was incorporated into the cockroach, and the distance that a virtual sensor could detect *d*_min_ was set as 0.1 times the normalized radius (4 cm). When the distance between a grid’s center point and an insect’s path is smaller than the perceptible distance *d*_min_, that grid is called the searched grid (grid_searched_).

The cockroach center of mass point (*x_c_*, *y_c_*) was obtained using a web camera from the image processing technique. The trajectory points traveled (route of cockroach) by the cockroach in the circular area are defined as *x_ci_* = [*x*_*c*1_ *x*_*c*2_ *x*_*c*3_ … *x_cm_*] and $y_{\mathrm{ci}}=\left[\begin{array}{ccccc} y_{c1} & y_{c2} & y_{c3} & \ldots & y_{\mathrm{cm}} \end{array}\right]$, where *m* is the number/length of points recorded through data acquisition on the developed UI. A 2-dimensional grid was generated using a nested for loop program in Python. The 2-dimensional grid point was sampled for 1/100 of circular radius arena expressed with *P*(*x*, *y*) = (*x_j_*, *y_k_*), *j* and *k* ranging from −100 to 100 with the length step of 1. The final values *P*(*x*, *y*) = (*x_j_*, *y_k_*) are divided by a constant of 100 for the normalized grid. A 2-dimensional grid was generated using Eq. 1. It will generate a normalized 2-dimensional grid from −1 to 1 on the *x* axis and *y* axis.

$$d_{\min}\left(j,k\right)=\min\left(\sqrt{{\left(x_{\mathrm{ci}}-\frac{x_{j}}{100}\right)}^{2}+{\left(y_{\mathrm{ci}}-\frac{y_{k}}{100}\right)}^{2}}\right.,\text{for}\;j=-100:100,k=-100:100$$ (1)

To find the number of the searched grid (grid_searched_), 0.1 was selected as the threshold. Equation 2 is applied to calculate the number of the searched grid. Any points *P*(*x*, *y*) with a distance of less than 0.1 from the cockroach route (*x_ci_, y_ci_*) will be set to 1, otherwise are assigned to 0.

$$\begin{array}{c} {\text{grid}}_{\text{searched}}=\sum_{i=1}^{m}d_{\min},d_{\min}\left\{\begin{array}{c} 1,d_{\min}<0.1\\ 0,d_{\min}\geq 0.1 \end{array}\right. \end{array}$$ (2)

The grid points from Eq. 1 are divided into 2 areas: inside and outside the circular area. Equation 3 calculates the total number of grid points inside the circular arena (grid_area_). On the basis of the calculation, the total number of grid points inside the circular arena is 31,397.

$$\begin{array}{c} \begin{array}{l} {\text{grid}}_{\mathrm{area}}=\sum_{j=-100}^{100}\sum_{k=\sum 100}^{100}d_{\mathrm{jk}},d_{\mathrm{jk}}\left\{\begin{array}{c} 1,d_{\mathrm{jk}}<1\\ 0,d_{\mathrm{jk}}\geq 1 \end{array}\right.\\ \;\text{where}\;d_{\mathrm{jk}}=\sqrt{{\left(\frac{x_{j}}{100}\right)}^{2}+{\left(\frac{y_{k}}{100}\right)}^{2}} \end{array} \end{array}$$ (3)

We propose a metric score, namely, search rate *ε*, to evaluate the cockroach movement performance in the circular bounded space. The search rate *ε* is calculated on the basis of the ratio of the number of grids searched within the experiment time to the overall number of grids inside a circular bounded space. The search rate is expressed in Eq. 4. In other words, an increase in the search rate means that a larger area can be searched, which can be said to be a more efficient search for the cockroach.

$$\varepsilon =\frac{{\text{grid}}_{\text{searched}}}{{\text{grid}}_{\text{arena}}}$$ (4)

To calculate the central rate *c_r_* and peripheral rate *p_r_* in Eqs. 6 and 7, the cockroach position points obtained from computer vision were computed using Eq. 5 to obtain the distance from the central point (0,0) or the center of the circular arena. A central rate of 1 means that the cockroach was at the central rate during the experimental time. It had never been at the peripheral rate and vice versa with the peripheral rate.

$$r=\sqrt{{x_{\mathrm{ci}}}^{2}+{y_{\mathrm{ci}}}^{2}}$$ (5)

$$c_{r}=\frac{1}{m}\sum_{i=1}^{m}r,r=\left\{\begin{array}{c} 1,r<0.9\\ 0,r\geq 0.1 \end{array}\right.$$ (6)

$$p_{r}=1-\left(\frac{1}{m}\sum_{i=1}^{m}r\right),r=\left\{\begin{array}{c} 1,r<0.9\\ 0,r\geq 0.1 \end{array}\right.$$ (7)

The normalized distance length traveled *d_t_* by the cockroach during walking movement, both free walking motion and stimulated walking motion in the circular arena, can be estimated using Eq. 8.

$$\begin{array}{c} d_{t}=\sum_{i=1}^{m}\sqrt{{\left(x_{c\left(i\right)}-x_{c\left(i-1\right)}\right)}^{2}+{\left(y_{c\left(i\right)}-y_{c\left(i-1\right)}\right)}^{2}} \end{array}$$ (8)

In this study, to increase the search rate score, the stopping time from the cockroach should be as small as possible. Therefore, the cockroach can move around the circular arena with a longer range by providing automatic stimulation when the cockroach is identified as a stop state. The stopping time *t_s_* during the experiment *t*_exp_ can be calculated using Eq. 9. The time required to stimulate the cockroach *t_st_* once in an experiment can be calculated using Eq. (10). We utilized a web camera as the ground truth for the cockroach state instead of the estimated state (stop/move) from the machine learning output.

$$\begin{array}{c} t_{s}\left(\text{second}\right)=\frac{m-\sum_{i=1}^{m}\text{state}\;}{m}t_{\exp}60,\text{state}=\left\{\begin{array}{c} 1,\text{move}\\ 0,\text{stop} \end{array}\right. \end{array}$$ (9)

$$\begin{array}{c} t_{\mathrm{st}}\left(\text{second}\right)=\frac{\sum_{i=1}^{m}\text{stim}}{m}t_{\exp}60,\text{stim}=\left\{\begin{array}{c} 1,\text{stimulation}\\ 0,\mathrm{no}\;\text{stimulation} \end{array}\right. \end{array}$$ (10)

## Proposed online machine learning

**Fig. 3.**
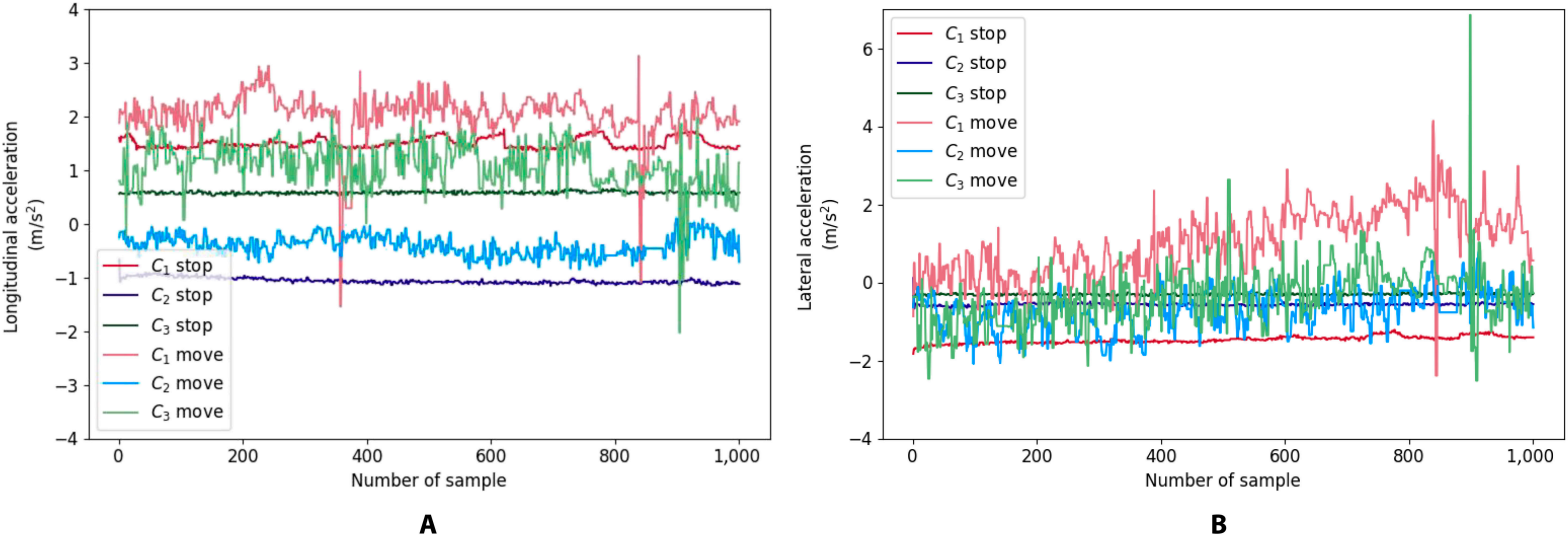
Accelerometer raw data. (A) Longitudinal acceleration. (B) Lateral acceleration.

The accelerometer measures linear acceleration due to motion and pseudo-acceleration caused by gravity. On the basis of our experiments, the bias/offset value for the lateral, longitudinal, and vertical accelerations will depend on the cockroach’s attitude angle (roll and pitch). The wireless backpack containing IMU was attached to the second segment of the cockroach thorax. The bias/offset resulting from the accelerometer varies among the cockroaches because of their size and the initial angles of the backpack. Three cockroaches [first cockroach (C_1_), second cockroach (C_2_), and third cockroach (C_3_)] were measured in 2 states, stop and move. The raw values of longitudinal and lateral accelerations are shown in Fig. 3A and B. The measured acceleration contained noise both in stop and move states. Noise increased when the cockroach was in a free-motion state. On the basis of this measurement, applying a constant threshold value as a simple control was difficult to distinguish between the stop and free motion states. The measured acceleration was noisy, and the motion of the cockroach was relatively slow. Moreover, the cockroach bent its body when the stimulation was provided to cerci. This motion contributed to more noise and oscillations of longitudinal and lateral accelerations.

The velocity of the cockroach could not be estimated directly from measured acceleration using IMU because of noisy measurement and bias. Drift will occur from numerical integration to estimate the velocity from acceleration measurement. It will be larger over time because of the accumulation of errors. Because of drift and bias, IMU is seldom utilized as a single sensor to estimate velocity and position. Previous research studies utilized sensor fusion to estimate velocity from IMU. Laser tracker and IMU were combined as sensor fusion to estimate the velocity of the robot end-effector in a previous study [25]. A low-cost IMU and global navigation satellite system were successfully applied to obtain a reliable vehicle velocity estimation [26]. Global navigation satellite system and IMU were utilized to estimate instantaneous velocity [27]. Other studies combined IMU with a camera [28], LiDAR [29], depth sensor [30], and ultra-wideband [31] to estimate state/attitude and minimize drift error. Sensor fusion has been proven for stable and reliable velocity or state estimation without drift.

In this study, because of the limited size of the backpack, we could not attach more sensors to the backpack for sensor fusion to estimate velocity. Furthermore, the measured acceleration and angular rate values contained noise and bias, which could change over time. We proposed a machine learning technique to process raw measured accelerometer and gyroscope without sensor fusion. Different cockroaches that were not included in real-time automatic stimulation experiments were used as training data. Machine learning was implemented to process noisy, drift, and biased signals from cockroach movement measurement.

### Feature extraction

The motion signals acquired through a wireless backpack are in the time domain and unsuitable for direct learning data. It is common to divide the time-series data into a collection of data called a window and extract the features for each window. A previous study reported that a window size of 1.5 s was sufficient for classifying MHC movements [20]. Therefore, this study extracted the features at intervals of 1.5 s. Statistical variance measures such as mean absolute deviation and interquartile range are widely used in IMU signal processing [32]. Skog et al. [33] also experimentally showed that the rotational kinetic energy obtained from the gyroscope information was particularly effective in determining whether the IMU was moving. The accelerometer signals were filtered using a first-order low-pass filter with the steady-state gain *K* = 1, and time constant *τ* = 0.1 as written in Eq. 11.

$$H\left(s\right)=\frac{K}{\tau s+1}$$ (11)

Ten time-domain features were chosen for the cockroach motion classification, as listed in Table 2. Mean, variance, skewness, kurtosis, range, mean absolute deviation, and interquartile range were extracted from the accelerometer (*a_x_*, *a_y_*, *a_z_*) and gyroscope (*g_x_*, *g_y_*, *g_z_*) signals. These features have a size of 42 extracted from the accelerometer and gyroscope signal. While the last 3 gyro energy features were extracted from the gyroscope signals (*g_x_*, *g_y_*, *g_z_*). The size of these features is 3. Each time-series signal from the accelerometer and gyroscope is denoted by *x*, while *L* is the number of signal data in one window *x* = [*x*_1_, *x*_2_, *x*_3_…*L*].

**Table 2.** Utilized time-domain features for movement identification of the cockroach.

Feature	Equation
Mean ($\bar{x}$)	$\bar{x}=\frac{1}{L}\sum_{i=1}^{L}x_{i}$
Variance (var)	$\mathrm{var}=\frac{1}{L-1}\sum_{i=1}^{L}{\left(x_{i}-\bar{x}\right)}^{2}$
Skewness (skew)	$\text{skew}=\frac{1}{\left(L-1\right)s^{3}}\sum_{i=1}^{L}{\left(x_{i}-\bar{x}\right)}^{3}$, where *s* is the standard deviation of *x*
Kurtosis (kurt)	$\text{kurt}=\frac{1}{\left(L-1\right)s^{4}}\sum_{i=1}^{L}{\left(x_{i}-\bar{x}\right)}^{4}$
Range (range)	range = max(*x*) − min(*x*)
Mean absolute deviation (MAD)	$\mathrm{MAD}=\frac{1}{L}\sum_{i=1}^{L}|x_{i}-\bar{x}|$
Interquartile range (IQR)	IQR = *Q*_3_ − *Q*_1_, where *Q*_3_ is 75th percentile *x* and *Q*_1_ is 25th percentile *x*
Gyro energy *x* axis	${\mathrm{GE}}_{x}=\frac{1}{L}\sum_{i=1}^{L}{g_{\mathrm{xi}}}^{2}$
Gyro energy *y* axis	${\mathrm{GE}}_{y}=\frac{1}{L}\sum_{i=1}^{L}{g_{\mathrm{yi}}}^{2}$
Gyro energy *z* axis	${\mathrm{GE}}_{z}=\frac{1}{L}\sum_{i=1}^{L}{g_{\mathrm{zi}}}^{2}$

### Machine learning

**Fig. 4.**
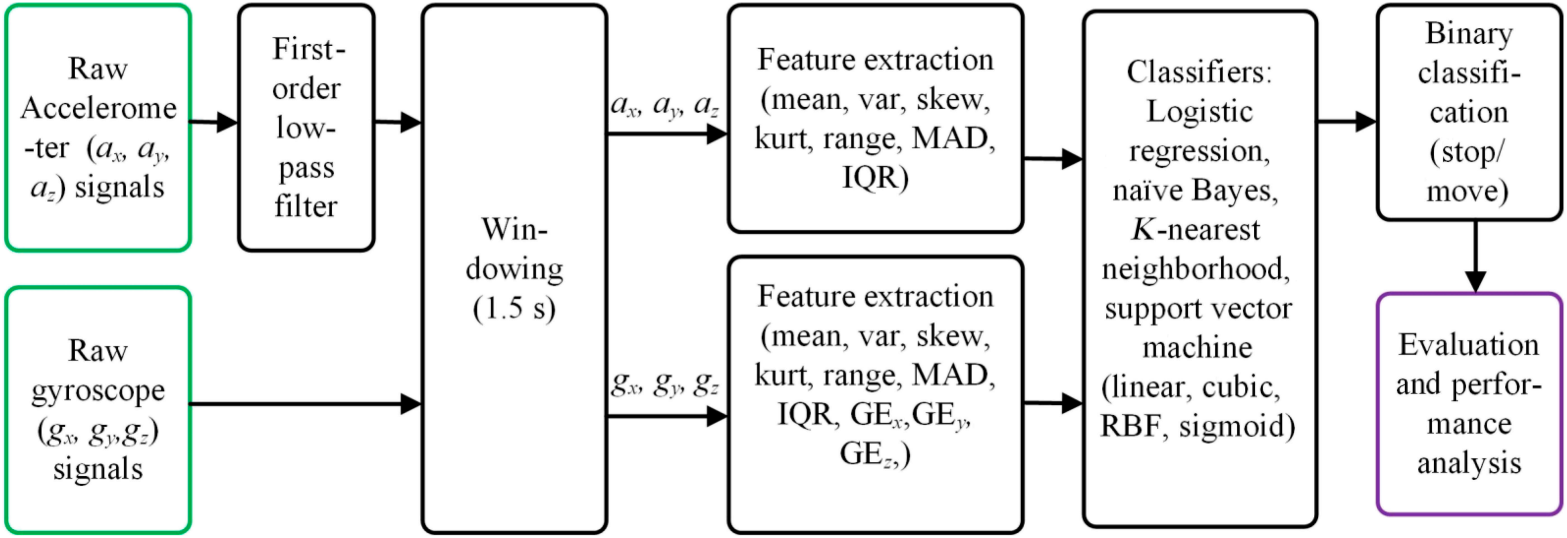
Block diagram of machine learning for cockroach motion identification. MAD, mean absolute deviation; IQR, interquartile range; GE, Gyro energy.

The overall block diagram for the applied machine learning process is summarized in Fig. 4. The raw signals were obtained wirelessly from an electronic backpack through a wireless transmitter connected via serial communication to the desktop PC. The signals were segmented using a window of 1.5 s or 30 data samples. In this study, logistic regression (LR), Naïve Bayes (NB), *K*-nearest neighbors (KNNs), and support vector machine (SVM) were implemented to classify the binary classification of the cockroach motion in the circular bounded arena (Stop/Move). LR, NB, and KNN were selected as the machine learning models for motion identification because they have no complex parameters and can be implemented in a large training dataset containing many features. These classifiers are suitable for this study since they have many extracted features from the accelerometer and gyroscope signals.

LR calculates a weighted sum of the input features, including bias, as expressed in Eq. 12. The LR for classification output the estimated probability $\hat{p}$ from a sigmoid function *σ* as written in Eq. 13. Where *X* is the instance features, while *β*_1_ and *β*_0_ are constants.

$$\sigma \left(X\right)=\beta_{1}X+\beta_{0}$$ (12)

$$\hat{p}=h_{\theta }\left(X\right)=\frac{1}{1+e^{-\left(\beta_{1}X+\beta_{0}\right)}}$$ (13)

The Eq. 13 output is a number between 0 and 1. The predicted value $\left(\hat{y}\right)$ from the LR model for the classification can be calculated using Eq. 14. On the basis of the sigmoid function, it predicts 1 if $\hat{y}$ is positive and 0 if $\hat{y}$ is negative.

$$\hat{y}=\left\{\begin{array}{c} 0\;\text{if}\;p<0.5\\ 1\;\text{if}\;p\geq 0.5 \end{array}\right.$$ (14)

NB is a simple and intuitive machine learning model for classification purposes. On the basis of Bayes’ theorem, the relationship between the class variable *y* and the dependent feature vector *X* = [*x*_1_, *x*_2_, .., *x_n_*] is expressed by Eq. 15.

$$P\left(y|x_{1},\ldots ,x_{n}\right)=\frac{P\left(y\right)P\left(x_{1},\ldots ,x_{n}|y\right)}{P\left(x_{1},\ldots ,x_{n}\right)}$$ (15)

The predicted class $\hat{y}$ from the NB can be calculated using Eq. 16.

$$\hat{y}=\arg\max_{y}P\left(y\right)\prod_{i=1}^{n}P\left(x_{i}|y\right)$$ (16)

In this study, the Gaussian NB algorithm was implemented for binary classification. The likelihood of the input features can be computed using Eq. 17.

$$\begin{array}{c} P\left(x_{i}|y\right)=\frac{1}{\sqrt{2{\mu \sigma }_{y}^{2}}}\exp\left(-\frac{{\left(x_{i}-\mu_{y}\right)}^{2}}{2\sigma_{y}^{2}}\right) \end{array}$$ (17)

KNN is relatively easy to implement in a real-time system and computationally efficient [23,24]. KNN has a fast computation in the training process. For the classification, KNN measures the similarity of the measured distance. Minkowski distance is a default distance metric in Python library [34]. The Minkowski distance between the classes/features *x_i_*, *y_i_* can be calculated using Eq. 18. When *c* is equal to 1 or *c* is equal to 2, the distance equation will be Manhattan distance or Euclidean distance, respectively.

$$d\left(x,y\right)={\left(\sum_{i=1}^{n}{\left|x_{i}-y_{i}\right|}^{c}\right)}^{\frac{1}{c}}$$ (18)

where *d*(*x*, *y*) is the distance between features (*x_i_*, *y_i_*), and *c* is a constant.

SVM has been extensively used as a machine learning for both regression and classification problems. For a 2-dimensional case, SVM computes linearly separable data that can be separated by a line/hyperplane. The can is expressed as in Eq. 19.

$$w^{T}x+b=0$$ (19)

where *w* is the weight vector and *b* is linear coefficient computed from the training data, while *x* is a new instance. After obtaining the hyperplane parameter, the predicted class $\hat{y}$ can be calculated using Eq. 20.

$$\hat{y}=\left\{\begin{array}{c} +1\;\text{if}\;w^{T}x+b\geq 0\\ -1\;\text{if}\;w^{T}x+b<0 \end{array}\right.$$ (20)

In this study, other kernels *K*(*x_i_*, *x_j_*) were used as classifiers, such as polynomial (cubic), radial basis function (RBF), and sigmoid kernels as expressed in Eqs. 21 to 23.

$$K\left(x_{i},x_{j}\right)={\left(\gamma \left(x_{i}x_{j}\right)+r\right)}^{d}$$ (21)

$$K\left(x_{i},x_{j}\right)=\exp\left(-\gamma {\left[x_{i}-x_{j}\right]}^{2}\right)$$ (22)

$$K\left(x_{i},x_{j}\right)=\tanh\left(\gamma \left(x_{i},x_{j}\right)+r\right)$$ (23)

All the classifiers mentioned above were implemented using Python programming. Scikit-learn library was chosen for building the machine learning model because it is a free, simple, and efficient tool for classification tasks. The constant and other parameters used in the machine learning models are presented in the Machine learning performance result section. The performance of the classifiers was evaluated using accuracy, precision, recall, and F1 score. The definitions of the evaluation metrics are expressed in Eqs. 24 to 27 using true positive (TP), true negative (TN), false positive (FP), and false negative (FN).

$$\text{Accuracy}=\frac{\mathrm{TP}+\mathrm{TN}}{\mathrm{TP}+\mathrm{TN}+\mathrm{FP}+\mathrm{FN}}$$ (24)

$$\text{Precision}=\frac{\mathrm{TP}}{\mathrm{TP}+\mathrm{FP}}$$ (25)

$$\text{Recall}=\frac{\mathrm{TP}}{\mathrm{TP}+\mathrm{FN}}$$ (26)

$$F1\;\text{score}=\frac{2\left(\text{Recall}\times \text{Precission}\right)}{\text{Recall}+\text{Precission}}$$ (27)

**Fig. 5.**
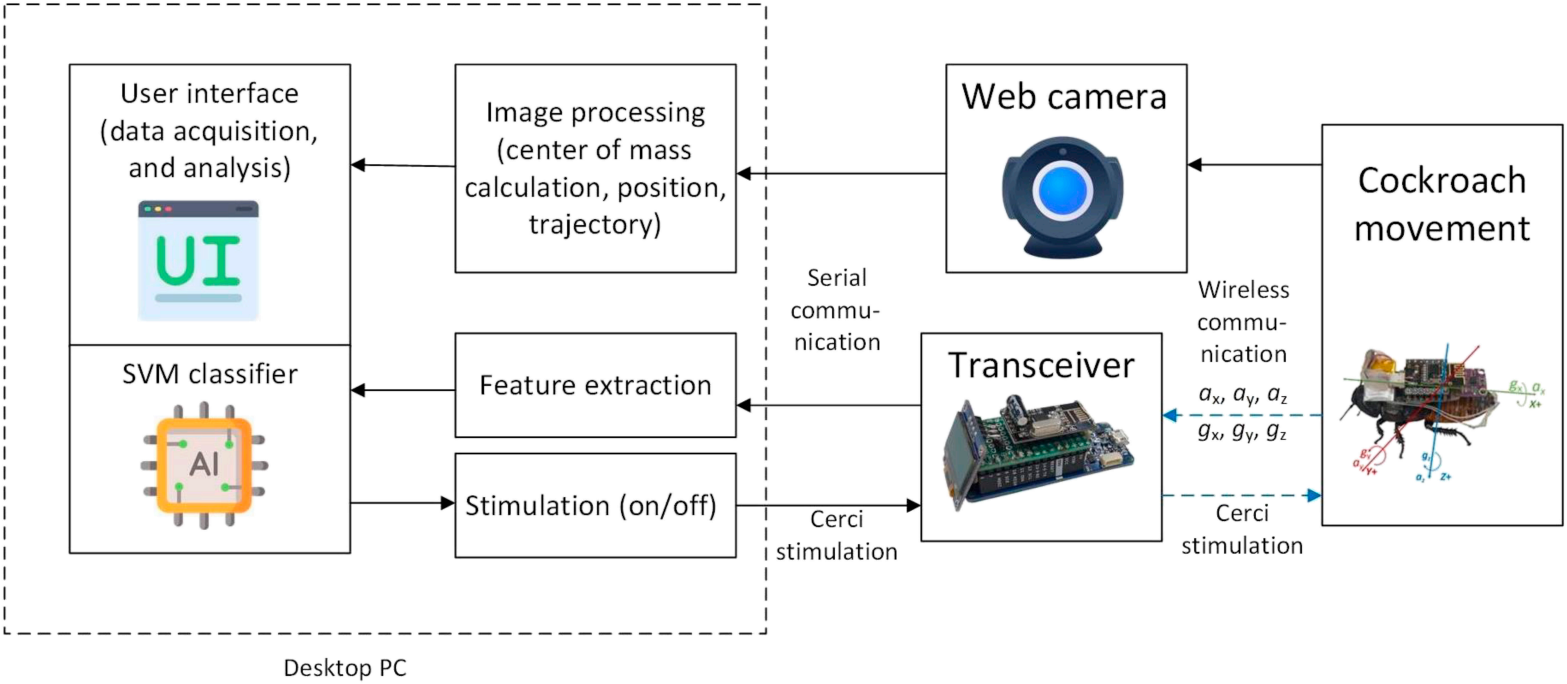
Implemented machine learning on the online classification and stimulation feedback for the cockroach.

In this research, after obtaining the performances of all-proposed classifiers, only the classifier with the highest performance was selected to be implemented on the cockroach system, as depicted in Fig. 5. The selected classifier performed an online binary classification task to automatically stimulate the cockroach’s cerci based on the obtained IMU data. For faster computation, the online classification task was carried out on the desktop high-performance PC instead of on the electronic backpack. If the classifier recognized that the cockroach stopped, then, after 1 s, the system sent a stimulation command to the cerci to trigger a free-walking motion on the cockroach.

## Results and Discussion

A previous study showed that the cockroach could autonomously follow the wall in a maze-like environment by providing electrical stimulation to the cerci [15]. In this study, cerci were stimulated to trigger the free-walking motion of the MHC. Our previous experiments showed that the cockroach followed the wall of the circular arena autonomously when the MHC was stimulated to the cerci.

### Machine learning performance result

For the machine learning as a classifier presented in the Machine learning section, all classifiers were implemented using Python programming. The classifiers were cross-validated using 10-fold cross-validation from all obtained and extracted dataset features for comparison performance. Cross-validation was conducted to ensure the validity of the classification results. The 10-fold cross-validation was performed using the *K*-fold cross-validator in the Scikit-learn library. The dataset for train/test indices was split into *k* consecutive folds by shuffling the dataset. Finally, the prediction accuracy and other metrics were computed by averaging the k prediction accuracy and other metrics.

The parameters were set to default for the binary classification task for the LR classifier. Gaussian distribution was implemented to calculate the likelihood of the input features for the NB classifier. In the KNN classifier, the number of neighbors *k* was selected equal to the square root of the training data sample size as performed by previous research [20]. Minkowski distance was implemented for the distance function, and *c* was set equal to 2. For the linear SVM, regularization parameter C was selected as equal to 1,000. For the polynomial and sigmoid kernels, the C parameter was set to 1 in this classification. The kernel coefficient of gamma *γ* was set to default in the cubic polynomial and sigmoid kernels. For the RBF SVM, the kernel coefficient *γ* and regularization parameters C were selected equal to 0.01 and 1,000, respectively.

**Fig. 6.**
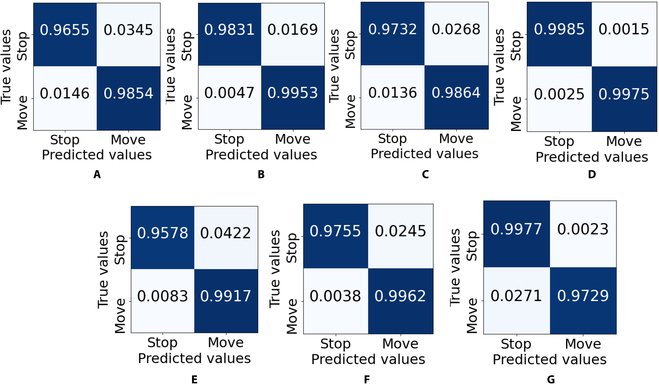
Normalized confusion matrix results. (A) LR. (B) NB. (C) KNNs. (D) Linear SVM. (E) Cubic SVM. (F) RBF SVM. (G) Sigmoid SVM.

**Fig. 7.**
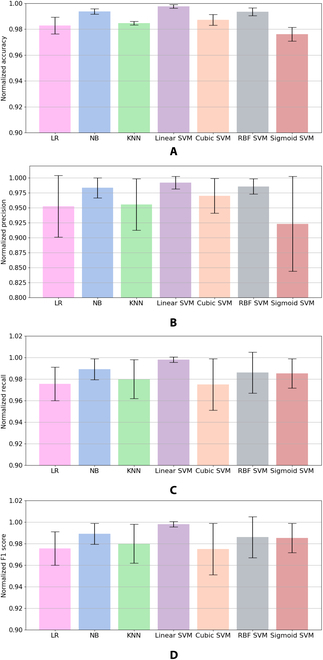
Normalized performance results. (A) Accuracy. (B) Precision. (C) recall. (D) F1 score.

The confusion matrix results for each classifier are presented in Fig. 6. The matrix is a normalized number from 0 to 1 in terms of accuracy in each class. The results show that accuracy in each class for all classifiers can reach more than 0.95 for binary classification. This number shows that all classifiers produce a high accuracy model of classifiers. Linear SVM produces the highest accuracy in both classes, 0.9985 for the stop class and 0.9864 for the move class. For other overall evaluation metrics for accuracy, precision, recall, and F1 score, linear SVM provides the highest performance metrics compared to other classifiers, as shown in Fig. 7. Therefore, a linear SVM was implemented for online cockroach automatic stimulation in the circular bounded space to maximize the search rate and traveled distance and minimize the stop time. In the previous study, linear SVM also provided the highest performance for the MHC motion mode identification compared to KNN, random forest, and linear discriminant analysis [20]. However, this previous study only focused on the offline classification of MHC motion.

### Free movement without machine learning stimulation

**Fig. 8.**
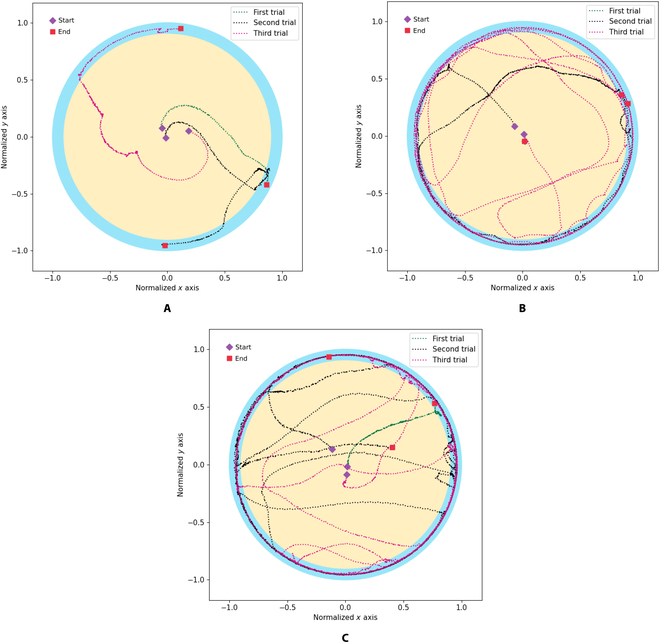
Cockroach trajectory without automatic stimulation. (A) First cockroach. (B) Second cockroach. (C) Third cockroach.

In this section, platinum electrodes were implanted on both cerci of cockroaches. Three male cockroaches were selected and used as cyborg cockroaches. Each cockroach was placed in the center of the circular arena for 7 min. The cockroach will be tested for free walking movement without cerci stimulation from the center of the central zone to the peripheral zone. The cockroach trajectory was processed and obtained using computer vision. The recorded trajectories for the 3 cockroaches are shown in Fig. 8. The starting points were indicated with purple rectangular points, while the end points were shown with magenta rectangular points. The test in the circular arena was repeated 3 times for Each cockroach. Each test was conducted for 7 min.

The movement performance metrics for the cockroaches were calculated using Eqs. 4 and 6 to 9 for computing search rate, central rate, peripheral rate, distance, and stop time, respectively. The performance metrics results are summarized in Table 3 for all cockroaches. The table shows that the third cockroach is the most active, and the first cockroach is the least active in the movement. On the basis of the experiment result without stimulation command, most of the cockroach positions in the cockroach test experiment were in the peripheral area. In addition, cockroaches tended to stay still and not move, especially in the first and second cockroaches. The average stop time of the first and second cockroaches are 354.55 and 210.95 s. The table shows that the third cockroach stop time average is 142.56 s, which can reach an average search rate of 0.48 and an average normalized distance of 36.40.

**Table 3.** Free movement of the cockroach.

Movement performance	First cockroach			Second cockroach			Third cockroach		
	1st trial	2nd trial	3rd trial	1st trial	2nd trial	3rd trial	1st trial	2nd trial	3rd trial
Search rate (*ε*)	0.10	0.15	0.20	0.01	0.39	0.72	0.07	0.70	0.67
Distance (*d_t_*)	5.47	9.56	12.63	7.63	18.54	46.00	10.75	50.23	48.23
Central rate (*c_r_*)	0.06	0.17	0.30	1.00	0.31	0.47	0.69	0.20	0.21
Peripheral rate (*p_r_*)	0.94	0.83	0.70	0.00	0.69	0.53	0.31	0.80	0.79
Stop time (*t_s_*)	392.02	342.99	330.02	420	126.85	86.01	379.41	20.48	27.78

### Movement with machine learning stimulation

**Fig. 9.**
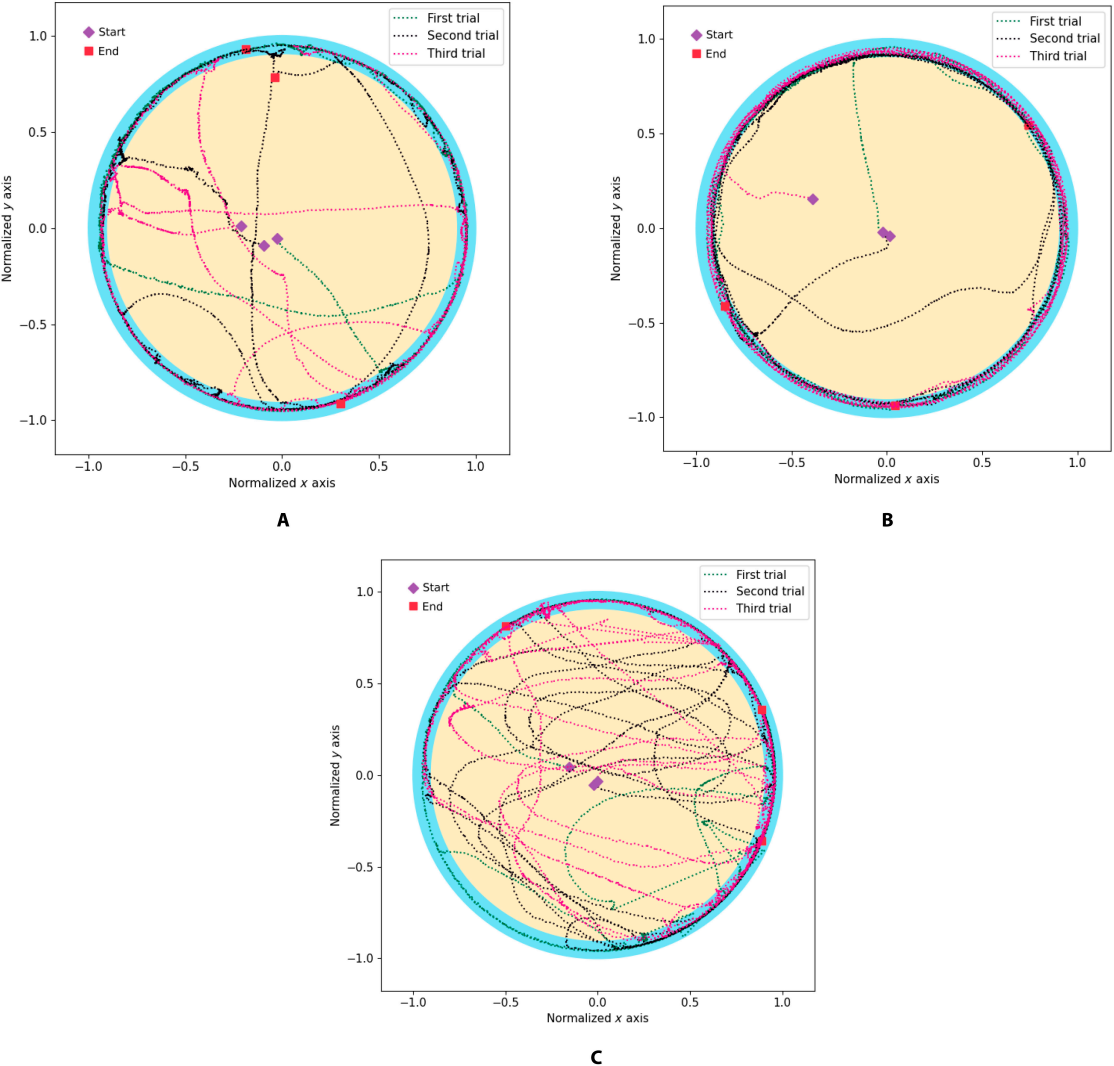
Cockroach trajectory with cerci stimulation from online SVM. (A) First cockroach. (B) Second cockroach. (C) Third cockroach.

In this section, 3 cockroaches were tested with automatic stimulation commanded from online linear SVM, as depicted in Fig. 5. When the SVM recognizes that the cockroach is in free-walking motion, the SVM will not send the electrical stimulation command from the desktop PC to the electronic backpack wirelessly through a wireless transceiver. If the SVM does not detect the cockroach’s free-walking motion (stop), then the system will provide electrical stimulation to the cockroach’s cerci. The stimulation signal on the cerci triggered the cockroach to make a free-walking motion. The stimulation signal was sent after the free-walking motion was detected by SVM for 1 s. Each test was repeated 3 times, and each test was carried out for 7 min. The result of automatic stimulation incorporating machine learning to optimize the cockroach movement performance is presented in Fig. 9.

The cockroach’s movement performance is summarized in Table 4. The third cockroach still was the most active. As seen in Fig. 9, when the cockroaches were in the peripheral zone, the cockroach autonomously followed the circular arena wall with and without automatic stimulation. This motion confirmed the previous studies for the German cockroach [24] and discoid cockroach [10] for free-walking motion without providing electrical stimulation. This study results confirmed that the cockroach performed wall following motion when the cockroach was in the peripheral zone, near the wall of the circular arena.

**Table 4.** Automatic stimulation movement of the cockroach.

Movement performance	First cockroach			Second cockroach			Third cockroach		
	1st trial	2nd trial	3rd trial	1st trial	2nd trial	3rd trial	1st trial	2nd trial	3rd trial
Search rate (*ε*)	0.44	0.59	0.62	0.41	0.51	0.37	0.53	0.80	0.83
Distance (*d_t_*)	27.37	32.166	33.17	24.39	37.02	40.18	34.60	48.95	74.29
Central rate (*c_r_*)	0.08	0.22	0.25	0.05	0.20	0.03	0.19	0.46	0.35
Peripheral rate (*p_r_*)	0.92	0.78	0.75	0.95	0.80	0.97	0.81	0.54	0.65
Stop time	112.53	87.49	52.12	11.26	11.26	10.39	118.40	11.36	33.20
Stimulation time (*t_st_*(*s*))	4.64	2.35	1.47	5.67	1.91	1.14	10.13	0.88	2.74

**Fig. 10.**
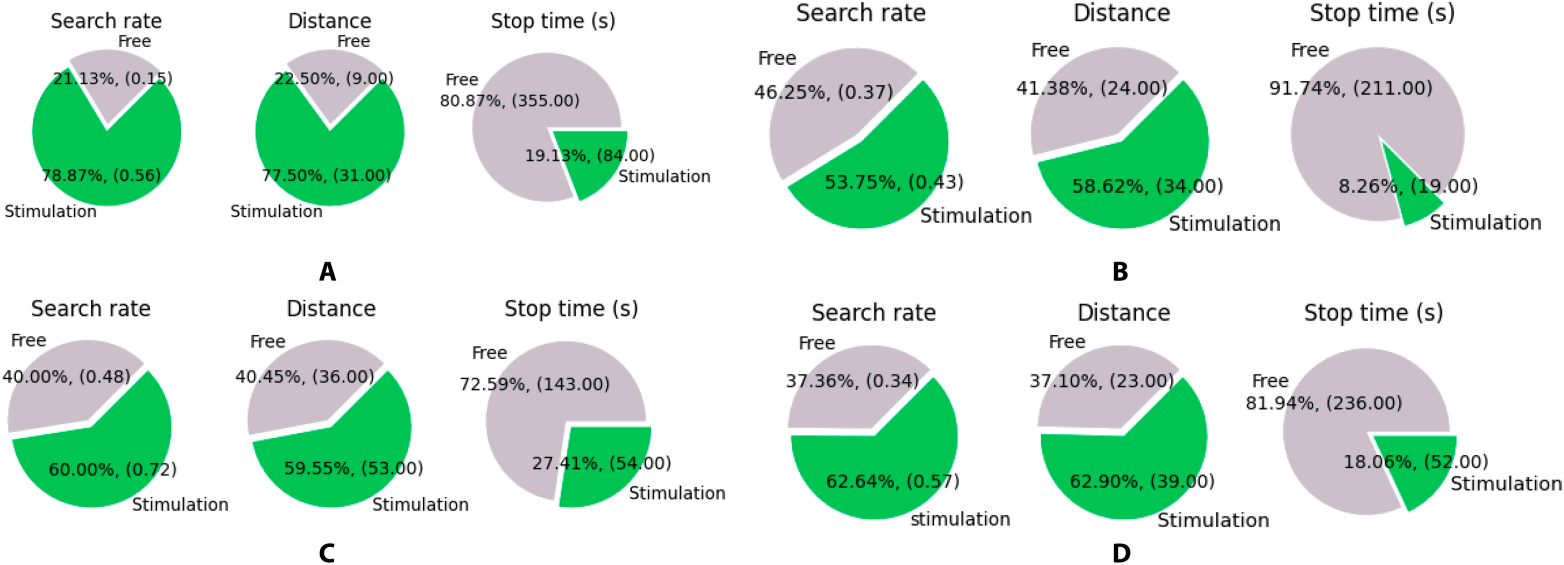
Performance of the cockroach. (A) First cockroach. (B) Second cockroach. (C) Third cockroach. (D) Overall cockroaches.

The movement performance metrics with automatic stimulation from machine learning and without automatic stimulation are plotted as presented in Fig. 10. The search rate and traveled distance improved by incorporating machine learning, and the stop time was reduced for each cockroach compared to the cockroaches without automatic stimulation. Overall, we can see that using online linear SVM to classify the cockroach motion and provide automatic stimulation to the cerci successfully optimizes the cockroach movements. The implemented linear SVM improved the search rate and distance traveled by cockroaches and reduced the stop time. The search rate and the distance were improved by 68% and 70% times, respectively, while the stop time was reduced by 78%. The average stimulation time for the first, second, and third cockroaches is 2.8, 2.9, and 4.6 s. The average stimulation time for all cockroaches is 3.4 s. The short stimulation time provided to the cockroach can reduce cockroach fatigue.

The proposed machine learning model successfully differentiated between stop and move states for 3 cockroaches that were not included in the training. On the basis of this result, the advantage of machine learning is robust and stable to sensor noise, bias, and initial backpack angle. The proposed method can be implemented for cyborg cockroaches to improve the movement performance of the cyborg cockroaches with minimal stimulation time. This proposed system is suitable for unstructured, under rubble, and unknown terrain exploration as well as search and rescue missions because the cyborg cockroaches have great and agile locomotion abilities. By implementing this system, the cyborg cockroaches can move without stopping because of their natural behavior, for example, stop moving in the dark or corner areas. Therefore, the explored area will be larger.

## Conclusion

We have successfully demonstrated the optimization for the movement performance of cyborg cockroaches in circular bounded space incorporating an online machine learning classifier. Accelerometer and gyroscope data received wirelessly from the cockroach motion through an electronic backpack and processed to extract 10-time domain features. On the basis of the offline classification performance result for the binary classification of the cockroach motion, linear SVM provided the highest performance compared to other classifiers. The linear SVM was applied to the developed UI with a multithreading algorithm running on the desktop PC. The online machine learning process did not implement on the electronic backpack due to many computations for the feature extraction. The applied linear SVM provided a stimulation command to the cerci after 1 s when SVM detected no movement on the cockroaches. By providing automatic stimulation on the cerci, the cockroaches could avoid obstacle/wall and follow the wall autonomously in the circular bounded space. The proposed system successfully increased the average search rate and traveled distance up to 68% and 70%, respectively, while the stop time was reduced by 78%.

## Data Availability

The data of this study are available from the corresponding author upon request.
